# Manipulation of intertrochanteric fractures in patients with below- or above-knee amputation using a fracture table: Two case reports

**DOI:** 10.1097/MD.0000000000024233

**Published:** 2021-01-15

**Authors:** Sang-Min Lee, Kuen Tak Suh, Young Kwang Oh, Won Chul Shin

**Affiliations:** a Department of Orthopedic Surgery, Pusan National University Yangsan Hospital; b Research Institute for Convergence of Biomedical Science and Technology, Pusan National University School of Medicine, Yangsan; c Professor Emeritus of Pusan National University School of Medicine; d Honorary Director of Orthopedic Surgery of Sehung Hospital, Busan, Korea.

**Keywords:** above-knee amputation, below-knee amputation, case report, fracture table, intertrochanteric fracture

## Abstract

**Rationale:**

In general, in the case of an intertrochanteric hip fracture, surgery is performed using a fracture table and by fixing the patient's foot to the boot piece. In patients with amputation of the affected lower limb, it is impossible to fix the foot to the boot piece; therefore, the traction and rotation of the fracture site cannot be maintained, leading to improper patient positioning. In such cases, a fracture table cannot be used intraoperatively to stabilize the fracture site. We report 2 cases of successful intertrochanteric fracture reduction using a fracture table for patients with below- or above-knee amputation.

**Patient's concerns:**

Both patients presented with left hip pain resulting from a fall.

**Diagnosis:**

Two elderly male patients with prior limb amputations below or above the knee presented with intertrochanteric hip fractures. Previous amputation of the lower limb on the same side of the fracture made it difficult to use a fracture table intraoperatively to stabilize the fracture site.

**Intervention:**

We performed fracture reduction using a modified fracture table for each patient. By altering the rotation of the boot piece and using additional skin traction bands, we could maintain proper patient positioning and rotation direction and obtain sufficient traction force.

**Outcomes:**

The chosen outcomes were fracture alignment and union at the end of follow-up and the ability to walk and perform activities of daily living. Reduction and intramedullary nail fixation using the fracture table were successful in both cases. Appropriate fracture union was achieved within 6 months, and the preoperative walking ability and activities of daily living were recovered in both patients, who were followed-up for 28 and 24 months.

**Lessons:**

Modification of the usual fracture table to suit patients with lower limb amputation helped us successfully perform intertrochanteric hip fracture surgery with the usual levels of traction and rotation required of the fracture site.

## 1 Introduction

The incidence of hip fractures is increasing due to the increasing age of the general population.^[1]^ The purpose of hip fracture treatment is to restore the preoperative gait status and activities of daily living by obtaining early union, early ambulation with rehabilitation, and reducing complications associated with fractures through appropriate fracture reduction and a successful surgical technique^[2,3]^; for this purpose, the fracture table is a widely used surgical device.^[3–8]^ In stable fractures, reduction is maintained only by axial traction and internal rotation in a slightly adducted position of the lower limb on the fracture table. In unstable fractures, anatomical reduction can be maintained by percutaneous or mini-incision techniques with proper traction and rotation on the fracture table.^[5]^

In most fracture tables, patients’ feet are tied to the boot piece, which provides accurate and stable traction control, and lower limb adduction, abduction, and external rotation.^[3,5,6]^ However, in patients with limb amputation on the same side, it is impossible to fix the foot to the boot piece; therefore, the traction and rotation of the fracture site cannot be maintained, leading to improper patient positioning.^[4–6,9–11]^

We report 2 cases of successful intertrochanteric fracture reduction using a fracture table to position patients with below- or above-knee amputation.

## 2 Case presentation

This study was approved by the institutional review board of Pusan National University Yangsan Hospital (05-2020-028). Written informed consent for publication was obtained from both patients.

## 3 Case 1

### 3.1 Patient information

The patient was an 80-year-old man who had left hip-joint pain after he slipped and fell. The medical history included chronic obstructive pulmonary disease, atrial fibrillation, and benign prostate hyperplasia. There was also a history of below-knee amputation due to squamous cell carcinoma of the left foot, but the patient was able to walk independently (independent community ambulation)^[12]^ and perform basic activities of daily living^[13]^ with his limb prosthesis.

### 3.2 Clinical findings

**Figure 1:**
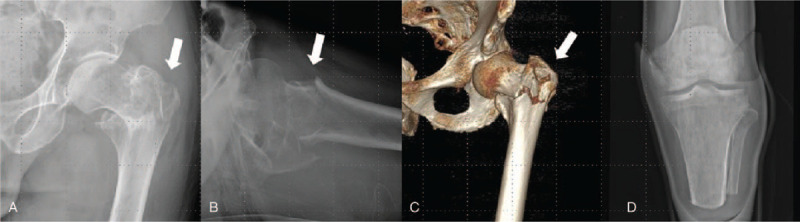
Case 1. (A) Plain radiographs of an 80-year-old man who fell and sustained an unstable left intertrochanteric hip fracture (white arrow, AO 31A3.3). (B) and (C) True lateral radiographs and 3-dimensional computed tomography image showing an unstable left intertrochanteric hip fracture. (D) Anteroposterior radiographs showing the below-knee amputation.

The type of fracture was A3.3 as per AO/OTA classification (Fig. 1). The patient was classified as stage III by the American Society of Anesthesiologists classification, according to the risk of preoperative anesthesia. The average dual-energy X-ray absorptiometry T-score of the femoral trochanter was –2.6, indicating osteoporosis.

### 3.3 Therapeutic intervention

**Figure 2:**
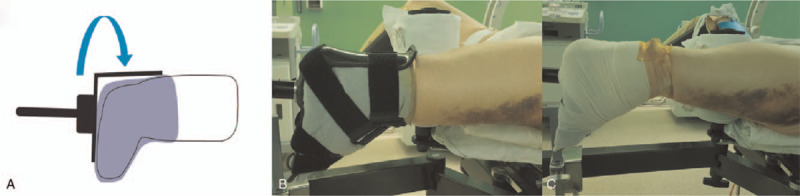
Case 1. (A) Schematic image showing an inverted boot applied to the below-knee amputation stump. (B) The photograph shows that proper fixation was obtained. (C) The drape should be appropriately placed, considering the incision.

Surgery was planned on the fracture table using a proximal femoral nail antirotation II (PFNA II, AO Synthes, Paoli, Switzerland). The patient underwent a reduction maneuver in the supine position, using a fracture table, under general anesthesia. The contralateral limb was placed at 90° of hip flexion and abduction, and fixed to the lower leg support device; C-arm image amplification fluoroscopy was easily accessible. On the affected limb, the stump was fixed with Velcro straps and elastic bandages, while the knee was flexed after inverting the boot piece upside-down (Fig. 2). In other words, by inverting the upper and lower parts of the boot piece, the flexed knee was fixed at the ankle joint position of the boot piece, which enabled traction and manipulation to be applied as in a usual femoral intertrochanteric fracture. Subsequently, fracture reduction was confirmed in anterior-posterior and cross-table lateral images, and sufficient traction and internal rotation were applied. The affected leg was fixed with approximately 10° to 15° of adduction to facilitate the insertion of the femoral nail, and a 3.2-mm guide pin was inserted into the tip of the greater trochanter. The pin position was confirmed in a lateral fluoroscopy amplified image. After the proximal reaming procedure, a 130° PFNA was inserted. Since the comminuted fracture extended to the lateral cortex, we used a 340-mm long nail. While maintaining reduction after the long nail insertion, another guide pin was inserted within 5 mm of the femoral head subchondral bone, and a spiral blade of appropriate length was chosen and positioned at the center of the femoral head in the anterior-posterior and cross-table lateral images; the apposition of the anterior cortex of the fracture site was confirmed in the cross-table lateral view after the insertion was completed.

### 3.4 Follow-up and outcomes

**Figure 3:**
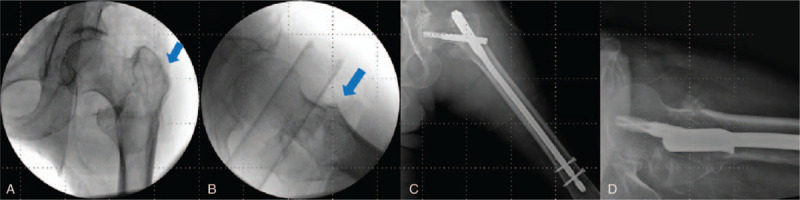
Case 1. (A) and (B) Intraoperative C-arm images showed proper traction and maintained reduction (blue arrow). (C) Postoperative radiographs showing adequate fracture reduction and fixation. (D) True lateral radiographs showed an apposition of the anterior cortex.

Postoperative plain radiographs showed appropriate fracture reduction and internal fixation (Fig. 3). The fracture was united at 6 months postoperatively; during the follow-up period of 28 months, there were no orthopedic complications, such as surgical site infection, as well as no systemic complications or deterioration of the underlying conditions. The patient performed total weight-bearing ambulation using the prosthetic leg and recovered his preoperative gait function^[12]^; he could also perform basic activities of daily living.^[13]^

## 4 Case 2

### 4.1 Patient information

An 89-year-old man was admitted to the hospital with left hip pain after a fall from a window. The left leg was amputated above the knee 50 years ago due to a trauma, and the patient had chronic stump pain and right knee osteoarthritis that resulted in limited walking ability (community ambulation with cane)^[12]^ and inability to perform basic activities of daily living^[13]^ with his limb prosthesis. There were no other underlying diseases.

### 4.2 Clinical findings

**Figure 4:**
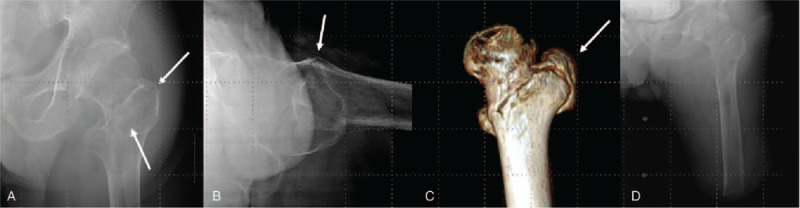
Case 2. (A) and (B) Preoperative plain radiograph images showing an intertrochanteric fracture in an 89-year-old patient with above-knee amputation (thin white arrow). (C) Three-dimensional computed tomography image showing a left intertrochanteric hip fracture. (D) Anteroposterior radiographs showing the above-knee amputation.

The type of fracture was A1.2, according to the AO/OTA classification, and the displaced fracture fragment formed an anterior angle (Fig. 4). The preoperative ASA classification was stage II, and the mean dual-energy X-ray absorptiometry T-score of the femoral trochanteric area was –3.7, indicating severe osteoporosis.

### 4.3 Therapeutic intervention

**Figure 5:**
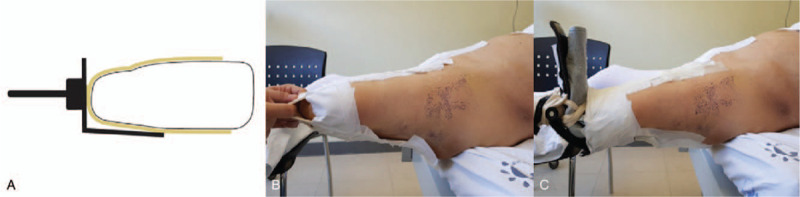
Case 2. (A) Schematic image showing a skin traction band and boot applied to the above-knee amputation stump. (B) Clinical photographs showing how the skin traction band was attached using both the anterior and posterior aspect of the thigh. (C) The back aspect of the boot piece (ankle part) supports the posterior aspect of the thigh; the band is also attached to the boot piece.

**Figure 6:**
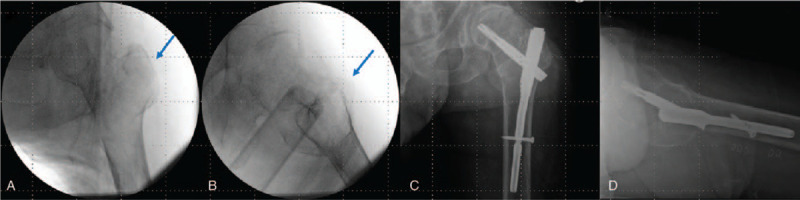
Case 2. (A) and (B) Intraoperative C-arm images showed that despite proper traction, complete reduction was not achieved and anterior angulation remained (thin blue arrow). (C) Postoperative radiographs showing adequate fracture reduction and proper fixation. (D) True lateral radiographs showing an apposition of the anterior cortex.

Surgery was planned using a PFNA II, and fracture reduction was performed in the supine position on the fracture table under general anesthesia. The stump of the affected side was fixed to the boot piece using a skin-pulling band. Unlike the usual procedure for skin traction, the band was not only fixed to the stump but also wrapped around the anterior and posterior thigh due to the aseptic drape and skin incision. The band was fixed using skin tape and surgical Ioban (3 M, Maplewood, MN); it was then fixed to the ankle part of the boot piece so that the lower side of the boot piece (ankle side) could support the stump along with the skin traction band (Fig. 5). Subsequently, sufficient traction and rotation could be applied. The state of reduction was evaluated by C-arm fluoroscopy, and it was confirmed that proper traction and internal rotation were obtained; however, anterior angulation of the fracture site remained. Anatomical reduction was performed using a kidney clamp through an incision, and internal fixation was performed using a standard 130° nail with a 200-mm length and 9-mm diameter (Fig. 6).

### 4.4 Follow-up and outcomes

Fracture union was achieved at 6 months postoperatively, and there were no fracture-related or general complications during the 2-year follow-up. Walking ability^[12]^ (community ambulation with cane) and activities of daily living were restored.^[13]^

## 5 Discussion

To date, there have been a few reports on the management of femur intertrochanteric fractures in patients with limb amputation, including below- and above-knee amputation.^[4,7,9,10,14]^ Patients with amputations are likely to lead active lives using limb prostheses.^[15,16]^ However, they may have a higher risk of fractures from slips or falls than the general population. Butler et al^[6]^ reported that 52% of patients with limb amputation experienced at least one slip in the previous year; moreover, the incidence of hip fractures is increasing with the increase in life expectancy. Femoral intertrochanteric fractures on the ipsilateral side of the limb amputation have specific problems related to patient positioning, surgical procedure, and using a fracture table, especially with a displaced fracture requiring reduction.

In previous studies, several methods for reduction of femoral intertrochanteric fractures in patients with limb amputation patients have been described (Table 1). First, a Steinmann pin is inserted into the distal femur or proximal tibia. The pin is then tied to a rope and traction applied to the boot piece.^[9]^ However, even though the Steinmann pin and rope method can maintain adequate traction and the abduction/adduction position, it is difficult to obtain rotational stability. The second method is to attach adhesive tapes and bands to the stump, similar to the skin traction method usually performed in children, and connect it to a fracture table.^[10]^ However, sufficient traction to the stump is more difficult to achieve through skin traction; additionally, there is a risk of adhesive tape and band loosening, which increases the risk of surgical field contamination and loss of reduction during surgery. There is also the possibility of stump and skin damage due to skin traction.^[8]^ A third option is to attach the external fixator to the distal femur and fix the rod of the external fixator to the fracture table.^[14]^ The fixation to the fracture table through the external fixator can provide sufficient traction and stability; however, it is difficult to make additional adjustments during surgery since the external fixator is connected to the fracture table; addressing this issue leads to additional medical costs. Invasive methods using a Steimann pin or external fixator increase the risk of stump soft tissue injury, pin site infection,^[17]^ and pull-out fracture due to traction being applied to an osteoporotic bone. Portnoy et al^[8]^ highlighted the risks of excessive traction, worsening of the wound when severe force is applied to the stump, and the risk of additional necrosis. They also reported that new scars may cause discomfort when using conventional prostheses. There is a method that enables the assistant to maintain reduction during surgery.^[7,18]^ This method is noninvasive and minimally harmful to the stump. However, it is difficult to obtain sufficient traction and torque, and maintain fracture reduction during surgery. In this context, the method used by us for the below-knee amputation patients is the same as when using a fracture table, without any additional procedure or invasive methods since the boot piece is inverted to fit the knee flexion and shape.^[4,7]^ Consequently, the procedure can be performed similarly to a general femur intertrochanteric fracture surgery, with no additional procedures required to obtain accurate reduction. Since the patella is located at the center of the boot piece, it is technically easy to perform reduction with internal or external rotation and adjust the reduction angulation. In addition, the possibility of skin damage and the risk of infection due to traction is reduced. However, this method is difficult to use when there is insufficient stump length or in patients with above-knee amputation. If the length of the stump is insufficient, the proximal portion of the boot piece may be moved higher to the surgical site. In this study, a skin traction band was used to fix the thigh to the boot piece for the patient with above-knee amputation. Since a rope was not used, unlike in the general skin traction method, the stump could be supported on the back of the boot piece, which allowed adequate torque to be obtained. In addition, the band is attached entirely to the anterior and posterior thigh, not just to the stump, minimizing the risk of adhesive tape and band loosening, reducing the risk of surgical site contamination, and loss of reduction during surgery.

**Table 1:** Methods of fractured limb support on the fracture table for patients with hip fracture and below-knee amputation.

	Reduction						
	Traction	Rotation	Maintaining reduction	Influence of long stump	Risk of infection	Risk of skin injury	Availability for AK amputation
Skeletal traction	Ο	×	Ο	Δ	Δ	Δ	Δ
Skin traction	Δ	×	Δ	Δ	Ο	Δ	Δ
External fixator traction	Ο	Ο	Ο	Δ	Δ	Δ	Δ
Assistant traction	Δ	Δ	×	Ο	Ο	Ο	Ο
Inverting boot traction	Ο	Ο	Ο	Ο	Ο	Ο	×
Modified skin traction	Ο	Ο	Ο	Δ	Ο	Δ	Ο

Assessment grade: Ο (good), Δ (fair), × (poor); AK = above-knee.

This study had some limitations. First, as this was a case report, there was a potential for selection bias. Second, the low number of cases should be kept in mind. Since a hip fracture has a large effect on the patient's quality of life, proper fracture reduction and choice of surgical procedure are crucial. The fracture table has been widely used in the treatment of hip fractures, but its application in patients with lower-limb amputations has limitations. In this case, we performed reduction, but fracture union was achieved using the fracture table for patients with below- and above-knee amputation. Clinical results were satisfactory.

To conclude, intertrochanteric fractures can be managed in patients with below-knee amputation by inverting the boot piece of the fracture table and applying it to the flexed knee and stump; additionally, in patients with above-knee amputation, fixation of the skin traction band on the anterior and posterior thigh as well as on the boot piece of the fracture table can be carried out safely and effectively. The creation of amputee-specific boot pieces may help make these adaptations unnecessary.

## Author contributions

**Correspondences:** Won Chul Shin.

**Data collection and analysis:** Sang-Min Lee, Kuen Tak Suh, Young Kwang Oh.

**Performed surgeries:** Kuen Tak Suh.

**Synopsis:** Sang-Min Lee, Kuen Tak Suh, Won Chul Shin.

**Writing the paper:** Sang-Min Lee, Won Chul Shin.

## Abbreviations

Abbreviation: PFNA = proximal femoral nail anti-rotation.
